# Artificial Intelligence-Based Anomaly Detection Technology over Encrypted Traffic: A Systematic Literature Review

**DOI:** 10.3390/s24030898

**Published:** 2024-01-30

**Authors:** Il Hwan Ji, Ju Hyeon Lee, Min Ji Kang, Woo Jin Park, Seung Ho Jeon, Jung Taek Seo

**Affiliations:** 1 Department of Information Security, Gachon University, Seongnam-si 1342, Republic of Korea; ilhwan1013@gachon.ac.kr (I.H.J.); 202240226@gachon.ac.kr (J.H.L.); 2 Department of Computer Engineering (Smart Security), Gachon University, Seongnam-si 1342, Republic of Korea; kmz0419@gachon.ac.kr (M.J.K.); shjeon90@gachon.ac.kr (S.H.J.); 3 Department of Software, Gachon University, Seongnam-si 1342, Republic of Korea; dsa1203ert@gachon.ac.kr; 4 Department of Computer Engineering, Gachon University, Seongnam-si 1342, Republic of Korea

**Keywords:** cyber security, anomaly detection, encrypted traffic

## Abstract

As cyber-attacks increase in unencrypted communication environments such as the traditional Internet, protected communication channels based on cryptographic protocols, such as transport layer security (TLS), have been introduced to the Internet. Accordingly, attackers have been carrying out cyber-attacks by hiding themselves in protected communication channels. However, the nature of channels protected by cryptographic protocols makes it difficult to distinguish between normal and malicious network traffic behaviors. This means that traditional anomaly detection models with features from packets extracted a deep packet inspection (DPI) have been neutralized. Recently, studies on anomaly detection using artificial intelligence (AI) and statistical characteristics of traffic have been proposed as an alternative. In this review, we provide a systematic review for AI-based anomaly detection techniques over encrypted traffic. We set several research questions on the review topic and collected research according to eligibility criteria. Through the screening process and quality assessment, 30 research articles were selected with high suitability to be included in the review from the collected literature. We reviewed the selected research in terms of dataset, feature extraction, feature selection, preprocessing, anomaly detection algorithm, and performance indicators. As a result of the literature review, it was confirmed that various techniques used for AI-based anomaly detection over encrypted traffic were used. Some techniques are similar to those used for AI-based anomaly detection over unencrypted traffic, but some technologies are different from those used for unencrypted traffic.

## 1. Introduction

The traditional Internet was designed based on transmission control protocol/internet protocol (TCP/IP) and was vulnerable to sniffing or spoofing attacks. As the Internet advanced, more information was exchanged across the network, including sensitive data such as intellectual property and business-related information. However, because TCP and IP do not include a functionality for protecting communication channels, such as encryption, by default, attackers could easily perform malicious behavior such as data theft or modification on the packets. These security threats have forced the Internet to use protected communication channels through security protocols such as transport layer security (TLS) [1]. As a result, unlike in the past, the proportion of encrypted traffic on the Internet is steadily increasing.

As the Internet environment is protected by encryption protocols, attackers also launch cyber-attacks through protected communication channels. According to a survey, attacks through encrypted channels continued to increase from 57% in 2020 to 80% in 2021, and by 2022, more than 85% of attacks carried out were encrypted [1]. The environmental approach, which uses deep packet inspection (DPI) to extract evidence of attacks from packets, is no longer valid [2]. Existing anomaly detection methods for network traffic include analyzing plaintext network traffic data and detecting anomalies based on payload analysis information within packets [3]. Since this anomaly detection method using key data in packets uses important data such as payload in plaintext traffic, it cannot be applied to anomaly detection performed on encrypted traffic in which important data are encrypted.

AI technologies, including machine learning and deep learning, provide feasible ideas for anomaly detection over encrypted traffic [4]. For this reason, research on anomaly detection over encrypted traffic based on AI is being actively conducted. Chen et al. [5] researched anomaly detection over encrypted traffic using traditional machine learning methods such as SVM and XGBoost. Bakhshi et al. [6] researched anomaly detection over encrypted traffic based on deep learning algorithms such as CNN and LSTM. Although research on AI-based anomaly detection over encrypted traffic is actively taking place, compared to this, there is not enough comprehensive reviewing of studies on AI-based anomaly detection over encrypted traffic.

Systematic literature review (SLR) is a research methodology that collects and evaluates existing research results and information on a specific topic in a systematic and standardized manner. In an SLR, research questions are set on a topic and a literature search, quality assessment, data extraction, result interpretation, and discussion are performed. Through this standardized analysis methodology, we can determine the current level of technology for the research topic and identify gaps in knowledge that have not yet been researched.

In this paper, we systematically review AI-based anomaly detection techniques over encrypted traffic. First, five research questions (RQs) are defined to set the scope of the SLR. Afterward, eligibility criteria are set to collect recent research from several bibliographic databases. Then, we select high-quality research through a screening process and quality assessment. A data extraction form is used to extract information to answer the previously set RQs from the selected literature. Finally, we organize and analyze the selected literature in terms of dataset, feature extraction, feature selection, preprocessing, anomaly detection algorithm, and performance indicators.

The composition of this paper is as follows. Section 2 presents a literature review of our AI-based anomaly detection over encrypted traffic. Section 3 presents our findings. Section 4 discusses the analysis results and Section 5 presents the conclusions and future research directions.

## 2. Methods

A systematic review is a type of secondary research that establishes straightforward RQs and examines, identifies, selects, and evaluates the literature using clear and iterative methods while minimizing bias. In this research, we performed a literature review on AI-based anomaly detection over encrypted traffic following the Preferred Reporting Items for Systematic Reviews and Meta-Analyses (PRISMA) [7] guidelines. The PRISMA checklist comprises seven sections (Title, Abstract, Introduction, Methods, Results, Discussion, Funding) and 27 topics. This ensures the overall quality of work by presenting tasks and requirements for each step of the literature review.

### 2.1. Research Questions

Before reviewing the literature, we set five RQs to analyze recent research on the review topic from a consistent perspective:

- RQ1. What datasets are mainly used to measure the performance of anomaly detection models for encrypted traffic, and what encryption method was mainly used to encrypt the data used in the experiment?
- RQ2. How can the features needed for AI model learning and anomaly detection over encrypted traffic be extracted?
- RQ3. What data preprocessing methods are commonly used in the earlier phases of the development of malicious activity detection algorithms?
- RQ4. What is the AI algorithm used to detect anomalies in encrypted traffic?
- RQ5. What are the performance indicators used to evaluate the performance of an anomaly detection model over encrypted traffic?

### 2.2. Eligibility Criteria

This step defines the adequacy that the literature must meet in order to identify recent research relevant to the RQs mentioned earlier, from multiple sources of information. The inclusion criteria for articles to be reviewed are as follows:

- Research written in English: Since English is the most dominant language used in modern medical, scientific, and engineering research, this ensures diversity in the literature to be reviewed.
- Research from conferences or journals that have passed peer review: This ensures the basic quality of the articles to be analyzed.
- The collected literature should cover data processing (feature selection and preprocessing) techniques for encrypted network traffic.
- The collected literature should cover anomaly detection techniques (algorithms) over encrypted network traffic.

However, even if it met the above criteria, studies corresponding to the concerns below were excluded from this review.

- Articles that present a methodology but do not have an objective evaluation of the proposal should be excluded.
- Studies outside the review topic, such as the classification of encrypted data rather than anomaly detection over encrypted traffic, should be excluded.
- Secondary research, such as survey-based studies, as opposed to primary research on data preprocessing and AI-based anomaly detection technologies for encryption traffic, should be excluded.
- Studies that conducted anomaly detection research only on data encrypted by encryption algorithms not currently in use due to design flaws or the development of alternative encryption algorithms should be excluded.

### 2.3. Information Sources

In this review, we collected articles published from 1 January 2019 to 31 August 2023 to analyze recent AI-based anomaly detection technologies over encrypt-ed traffic. To conduct reliable research and analysis of articles, we conducted a literature review of publications from Web of Science (WoS), a citation index database provided by Clarivate Analytics, and Scopus, a global academic literature database platform created in 2004 by Dutch publisher Elsevier. Additionally, the additional literature search was conducted using Goggle Scholar, ACM Digital Library, a comprehensive bibliographic database focused on computing, and IEEE Xplore Digital Library, which provides reliable research publications such as journals, conferences, standards, e-books, and curriculums.

### 2.4. Search and Study Selection

To select articles to be analyzed in this research, the search queries used were selected based on our experience and expertise. When selecting keywords included in the query, we selected keywords to search for articles that were not limited to a specific data type or environment and had a broad scope of research use. For example, research utilizing data generated by private cryptographic protocols used only in specific environments were excluded from this review. Based on these considerations, we created queries centered around keywords such as AI, anomaly detection, and encrypted traffic. This criterion is not limited to article searches but is also used in the subsequent article selection stage. Table 1 shows the queries used to collect the literature from each bibliographic database.

The selection of articles to analyze was performed through the following process:

1. Articles identified in the bibliographic database using the search query are transferred to the reference management software EndNote 21.
2. The same articles retrieved from different bibliographic databases are removed.
3. Articles that present a methodology but do not have an objective evaluation of the proposal should be excluded.
4. Identified articles will be reviewed based on the title and abstract according to the above eligibility criteria.
5. To determine which documents should be included in this review, a full-text evaluation of the remaining documents is performed through steps 2 to 4.

We carried out the above process based on our roles, and several meetings and discussions were held at each stage. Two reviewers worked independently to identify the literature that met the inclusion criteria. Two other reviewers reviewed abstracts and full-text articles to evaluate the suitability of the identified literature. Finally, two reviewers evaluated the suitability of the selected articles using a quality assessment tool.

### 2.5. Data Collection Process

In this review, we developed a data extraction form and collected data from each article according to this form. Additionally, to more flexibly extract data from each article, evidence-based software engineering (EBSE) guidelines were used to develop a data extraction form [8]. This form contains various information, including author, affiliations, publication date, journal/conference, IF, journal-level quartiles, dataset, feature selection, features, preprocessing techniques, classification algorithm, and experiment results. Throughout the process, to minimize data extraction bias, two authors extracted data from the literature, and the remaining authors cross-validated it.

### 2.6. Quality Assessment

In this article, cross-validation between authors was carried out to control the author’s bias regarding the literature to be reviewed. If bias is included in the literature review, the reliability of the research results may be reduced, and incorrect conclusions may be drawn. Although a systematic literature review must provide objective and fair answers to RQs, if bias is not removed through appropriate methods, certain research may be over- or underestimated. For this reason, cross-validation between authors was performed, and this process confirmed whether the following content was included in the literature.

1. Review topic. Research should suggest ways to detect anomalies or attacks in traffic.
2. Contextual information. Sufficient contextual information must be provided to interpret the results.
3. Data. The research article must provide a detailed description of the data used in the experiment. This affects the reliability of the research results. This is also essential to answer research question RQ1.
4. Details. Accurately conveying the data processing method and normal/anomaly classification explanation proposed by the research helps us to answer research questions RQ2 to RQ4.
5. Experimental results. Experimental results play an essential role in proving the validity of the research.

## 3. Results

### 3.1. Study Selection

**Figure 1:**
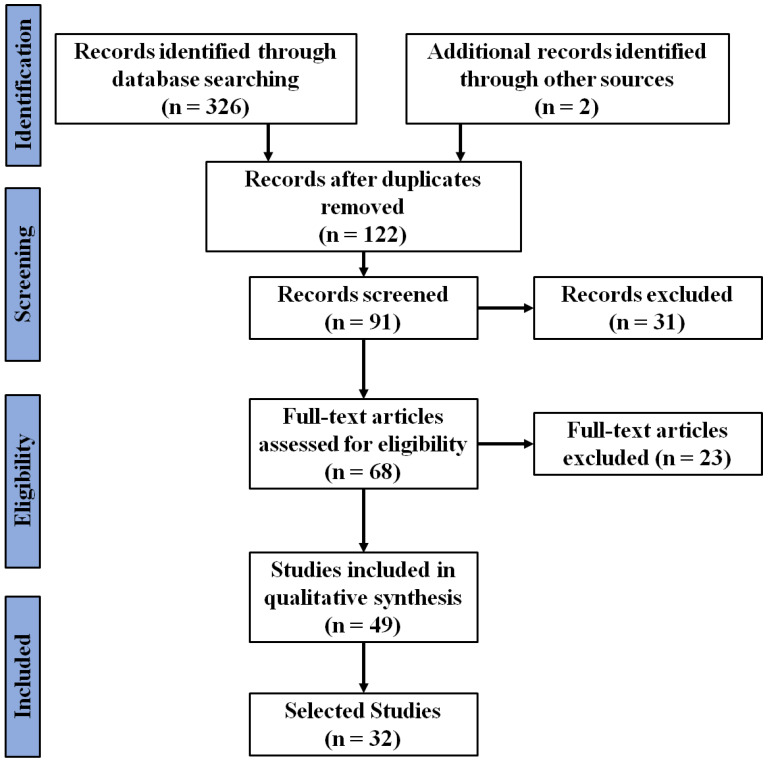
PRISMA flow chart.

A total of 328 articles on AI-based anomaly detection and encrypted traffic analysis were identified from the Web of Science, Scopus, ACM digital library, IEEE Xplore digital library and Google Scholar literature databases from 1 January 2019 to 31 August 2023. Among these, 56 articles from Web of Science, 89 articles from Scopus, 121 articles from ACM digital library, 60 articles from IEEE Xplore digital library, and 2 articles from Google Scholar (direct search) were selected. Among these, 206 duplicate articles were removed using EndNote 21. A total of 31 articles were excluded through title and abstract screening, and an additional 23 articles were removed through a full-text evaluation. A total of 49 articles were considered for quality assessment, and finally, 32 articles were included in this review through a consensus meeting applying the eligibility criteria. The detailed screening process is shown in Figure 1.

### 3.2. Summary of the Identified Literature

Table 2 summarizes the reviewed literature. “Dataset” describes the type of dataset used in each article, and “Encryption protocol” indicates how the data used in the research are encrypted. “Feature extraction” describes extracting features for AI-based anomaly detection over encrypted traffic. “Feature selection” expresses a feature selection method to improve AI-based anomaly detection performance. “Preprocessing” is a feature preprocessing method to improve AI-based anomaly detection performance. Lastly, “Classification algorithms” represent AI algorithms for classifying normal and anomaly-encrypted traffic.

### 3.3. Study Characteristics

This section describes the characteristics of the research included in this review based on the information extracted in Section 2.5. Table 3 shows the number of articles this review includes by publication year and reference type. The quality assessment results show that the number of journal articles included in this review is better than that of conference articles. Journal sources were selected to cover diverse research areas such as computer science and cybersecurity.

As can be seen in Table 3, the number of articles published on anomaly detection over encrypted traffic is increasing. It is expected to increase depending on the importance of the research.

**Figure 2:**
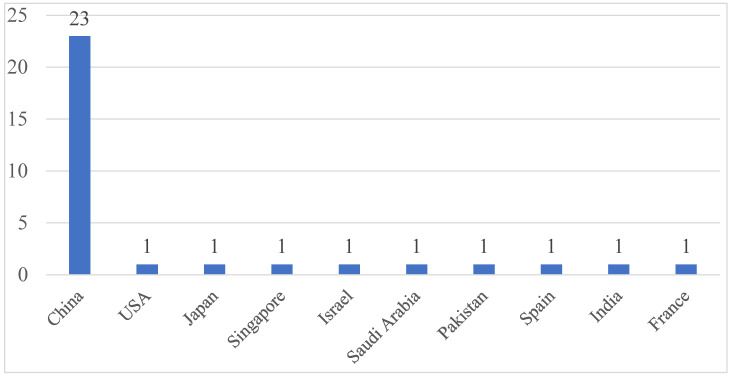
The number of articles in each country.

Figure 2 shows the number of publications by country for the literature included in this review. As shown in this figure, it has been confirmed that China is conducting more research than other countries in the field of anomaly detection over AI-based encrypted traffic. This suggests that China is contributing a lot to cybersecurity in research on anomaly detection over encrypted traffic.

### 3.4. Dataset

With machine learning and deep learning development, large amounts of data are required to learn models. The model learns patterns inherent in the data. Therefore, if the dataset is small or biased, the model’s generalization performance is poor and cannot be evaluated correctly. For models to detect anomalies in encrypted traffic, the dataset used for evaluation is important in order to maximize detection performance and ensure versatility. We included the datasets used by each study in the article analysis. Therefore, articles that used unpopular datasets (i.e., encrypted traffic with the deprecated cryptographic algorithms) or for which the dataset (i.e., encrypted traffic with private cryptographic algorithms) could not be identified were excluded from this review. The dataset used in each article is identified in the “dataset” column of Table 2. Additionally, we describe a summary of the datasets used by each analyzed article to answer RQ1.

The CTU-Malware-Captures dataset is frequently used in cybersecurity research and was developed to capture and analyze network traffic related to botnets [9]. This dataset includes network traffic in the environment of command and control (C2) communications, data exfiltration, malware propagation, and botnet-related malicious activity. The dataset consists of labels corresponding to the identified botnets’ behavior to facilitate data analysis and detection. However, because this dataset was created to detect anomalies based on general network traffic, it contains a relatively large amount of plain text traffic. Therefore, for AI-based anomaly detection research over encrypted traffic, SSL/TLS-related traffic must be separately extracted from the dataset. The CTU-Malware-Captures dataset was developed in 2013, and was updated until 2018. Furthermore, the dataset contains more than 3% encrypted traffic data.

The ISCX VPN-NONVPN dataset is developed to distinguish network traffic generated by a virtual private network (VPN) from that of a non-VPN [13]. In order to collect network traffic generated from a VPN and non-VPN in ISCX, network traffic generated from a VPN and network traffic generated from a non-VPN were captured by targeting services such as Skype and Facebook. Because the ISCX VPN-NONVPN dataset is mainly used to classify network traffic as VPN or non-VPN, it does not include data on cyber-attacks, but is combined with cyber-attack-related datasets collected in other VPN environments. It is used for anomaly detection system training and performance evaluation. The ISCX VPN-NONVPN dataset was developed in 2016, and was updated until 2021. Additionally, this dataset contains approximately 10.24% encrypted traffic data.

The CTU-13 dataset is a network traffic dataset in which various behaviors are captured, including normal traffic and malicious traffic [20,59]. This dataset is designed to support the evaluation of intrusion detection models and consists of over 100,000 encrypted traffic flows from seven well-known types of malware such as dridex and trickbot. This dataset is not designed to detect anomalies in cryptographic protocols and therefore contains a relatively large amount of non-encrypted traffic. However, since SSL/TLS encryption protocol traffic is included in the dataset, a sub-dataset to be used to evaluate an anomaly detection model over encrypted traffic can be created by extracting encryption protocol-related data. The CTU-13 dataset was developed in 2013, and was updated until 2015. Also, the dataset contains approximately 2.29% of encrypted traffic data.

The USTC-TFC 2016 dataset consists of normal traffic and traffic caused by malicious code [14]. This dataset includes 10 types of traffic generated by website malware in real network environments from 2011 to 2015. In addition, normal traffic includes 10 types of traffic collected using IXIA BPS. The USTC-TFC 2016 dataset was developed in 2019 and is available to date. Additionally, this dataset contains approximately 2.02% encrypted traffic data.

The NSL-KDD dataset was proposed to solve several problems, such as duplicate records in the KDD 99 dataset [15,60]. Because NSL-KDD lacks data from network IDS, it may not perfectly represent the real-world network. However, because it contains data on various types of attacks and intrusions, including denial of service (DoS), it can be used to classify types of intrusion detection attacks. Therefore, if NSL-KDD is used, evaluation results of various research, such as intrusion detection and classification of intrusion detection types, can be derived. The NSL-KDD dataset was developed in 2019 and is available to date. Additionally, this dataset contains approximately 3.85% encrypted traffic data.

The UNSW-NB15 dataset was created by generating raw traffic for actual normal behavior and synthetic attack behavior using the IXIA PerfectStorm tool [16]. This dataset includes nine attack types: fuzzers, analysis, backdoor, DoS, exploits, generic, reconnaissance, shellcode, and worms. The dataset contains more than 2 million records, which are provided in PCAP, BRO, Argus, and CSV files [61]. UNSW-NB15 dataset was developed in 2015, and was updated until 2021. Furthermore, the dataset contains more than 2% encrypted traffic data.

The CIC-IDS-2017 dataset was developed to address the challenge of upgrading the performance of anomaly detection models due to the lack of reliable datasets to test and validate IDS and IPS [17]. CIC-IDS-2017 includes network traffic generated by regular and cyber-attacks. Records in the dataset also include labeled data based on timestamp, source and destination IP, source and destination port, protocol, and cyber-attack. Implemented attacks include Brute Force FTP, Brute Force SSH, DoS, Heartbleed, Web Attacks, Aggregation, Botnet, and DDoS. The dataset includes encrypted and unencrypted traffic, including HTTP, HTTPS, FTP, and SSH [62]. The CIC-IDS-2017 dataset was developed in 2017, and was updated until 2019. Additionally, this dataset contains approximately 2.9% encrypted traffic data.

The Datacon 2020 dataset is a dataset on the detection of encrypted traffic caused by malware and was released through the DATACON2020 competition. The traffic included in this dataset originated from malicious and benign software using port 433 (i.e., TLS) from February to June 2020. Malicious traffic was collected from malware running in Qianxin Lab’s Skydome sandbox, and the converse was from legitimate software [23]. Datacon 2020 dataset was developed in 2020 and is available to date. Additionally, this dataset contains approximately 7.92% encrypted traffic data.

The Malware-Traffic-Analysis.net (MTA) dataset is a publicly available dataset for studying network traffic of malware [10]. Among the network traffic collected from June 2013 to the present, network traffic collected from January 2018 to January 2022 was selected, and eight popular types of malware (dridex, hancitor, emotet, icedid, qakbot, ursnif, zeus, and trickbot) were employed to create the dataset. The MTA dataset was developed in 2013, and was updated until 2023. Also, each source traffic pcap file in the dataset contains 0% to 20% encrypted traffic data, and dataset contains more than 190,000 encrypted session data.

The stratosphereips (STRA) dataset is a dataset created by capturing network traffic generated by actual malicious code [35]. This dataset contains network traffic caused by various types of malwares, including zeus, yakes, cridex, cerber, artemis, dynamer, and ursnif. The STRA dataset was developed in 2013, and was updated until 2018. Additionally, this dataset contains approximately 2.5% encrypted traffic data.

Jason Stroschein’s public Github malware dataset is a comprehensive dataset containing malicious traffic disclosed by Jason Stroschein on Git. Jason Stroschein’s public Github malware dataset includes traffic samples generated by malware, including trickbot [38]. Jason Stroschein’s public Github malware dataset was developed in 2020, and was updated until 2023. Also, the dataset contains approximately 8.92% of encrypted traffic data.

The UNSW NS 2019 dataset contains encrypted normal and malicious traffic collected from over 28 different IoT devices. However, features extracted from captured raw packets are not provided [43]. The UNSW NS 2019 dataset was developed and released in 2019 and is available to date. The MCFP dataset was developed in 2013, and was updated until 2018. Additionally, this dataset contains approximately 25.1% encrypted traffic data.

The CIC-AndMal2017 dataset was developed to detect Android malware and includes network traffic samples caused by malware [31,63]. The dataset includes 200,000 normal samples, 200,000 malware samples, 400,000 traffic, 14 major malware categories, and 191 major malware groups. The CIC-AndMal dataset includes the following types of malwares: adware, backdoor, file infector, no category, Potentially Unwanted Apps (PUA), ransomware, riskware, scareware, trojan, trojan-banker, trojan-dropper, trojan-sms, trojan-spy, and zero-day. The CIC-AndMal2017 dataset was developed in 2018, and was updated until 2019. In addition, over 25% of this dataset consists of encrypted traffic data.

The Malware Capture Facility Project (MCFP) dataset was developed to capture and analyze long-term network traffic caused by malware [33,59]. This dataset consists of normal network traffic and network traffic generated by 13 types of malwares. The MCFP dataset was developed in 2013, and was updated until 2018. Additionally, this dataset contains approximately 2.5% encrypted traffic data.

The CICIDS-2012 dataset is a dataset that includes both normal network traffic and network traffic generated by malicious actions [27]. Various multi-stage attack scenarios were employed to generate the dataset. The cyber-attacks used to create the dataset are largely divided into four categories (distributed denial of service (DDoS), brute force SSH, infiltrating transfer, and HTTP DoS), and HTTP DoS, and network traffic in HTTP, SSH, SMTP, POP3, IMAP, and FTP communication environments caused by cyber-attacks is included in the dataset. The CICIDS-2012 dataset was developed in 2012, and was updated until 2019. Additionally, this dataset contains approximately 1.06% encrypted traffic data.

The CIC-InvesAndMal2019 dataset includes static functions and API call permissions and intents, and all generated log files include all log files generated by dynamic functions in three stages (during installation, before restart, and after restart) [47]. Malware samples used to create the CIC-InvesAndMal2019 dataset include adware, ransomware, scareware, and SMS malware. The CIC-InvesAndMal2019 dataset was developed in 2019 and is available to date. In addition, over 25% of this dataset consists of encrypted traffic data.

The CIRA-CIC-DoHBRW-2020 dataset was created to perform anomaly traffic classification model and evaluation for DNS-over-HTTPS (DoH) [49,64]. The researchers deployed DoH within the application in a two-tiered approach to detect and characterize DoH traffic using a time series classifier. The dataset includes malicious and normal DoH traffic, and tools such as Google Chrome, Mozilla Firefox, dns2tcp, DNSCat2, and Iodine were used to capture traffic. Additionally, AdGuard, Cloudflare, Google DNS, and Quad9 were used as servers to process DoH requests. The CIRA-CIC-DoHBRW-2020 dataset was developed in 2020 and is available to date. Furthermore, the dataset contains more than 30% encrypted traffic data.

The CES-CIC-IDS 2018 dataset is a dataset that includes both normal network traffic and network traffic generated by malicious activities. The dataset includes data preprocessed using CICFlowmeter for traffic generated by Brute-force attack, Heartbleed attack, Botnet, DoS, DDoS, Web Attacks, and a cyber-attack via infiltration of the network the from inside [58]. The CES-CIC-IDS 2018 dataset was developed in 2018 and is available to date. Furthermore, the dataset contains more than 25% encrypted traffic data.

### 3.5. Feature Extraction

The method of extracting features necessary for learning AI models and detecting anomalies from encrypted traffic is different from the method of extracting features from existing plain text traffic. This section summarizes feature extraction techniques frequently used to extract features for performing AI-based anomaly detection over encrypted traffic. We describe a summary of the feature extraction used in the research to answer RQ2.

- Statistics-based feature extraction

Statistics-based feature extraction refers to a statistical method used to summarize and explain the main features and patterns of a dataset. Generally, information such as data distribution, central tendency, and volatility are used as statistical features. Unlike plaintext traffic, encrypted traffic does not contain a wealth of information that can be intuitively identified without decryption. However, due to a general network environment in which the key used to encrypt data is unknown, most research performs anomaly detection by relying on identifiable information and statistical characteristics in network traffic without decryption. For this purpose, information such as source IP address, destination IP address, source MAC address, destination MAC address, protocol type, and statistical processing functions for this information are mainly used. The procedure for collecting these characteristics is roughly as follows. First, raw network traffic data, including encrypted packets, is collected and the following basic data rectification is performed on it: duplicate packet removal, missing value handling, timestamp-based sorting, and packet filtering. Then, statistical features are extracted from the remaining packets through statistical processing, mainly producing the following features: flow duration, packet count, byte count, packet size statistics, packet transfer time statistics, protocol distribution, and port distribution. Because these characteristics are not information included in the packet payload, they do not provide direct evidence of a cyber-attack. However, these features can be used to train or verify anomaly detection models by providing abstract information about network flows. The remainder of this section describes frequently used tools and open sources for extracting statistical features from network packets.

CICFlowMeter is an open source designed for extracting statistical-based features from raw packet data [65]. CICFlowMeter captures statistics on various traffic flows such as packet count, byte count, duration, and packet transfer time statistics [66]. Additionally, the tool calculates statistics such as mean, median, and standard deviation for the preceding features to provide a distribution of data sizes within the encrypted flow. In addition, CICFlowMeter considers the distribution of packet lengths and produces features such as average packet length, change in packet length, and entropy. Various research has proposed anomaly detection models over encrypted traffic using statistical features extracted by CICFlowMeter [20,27,28,29,30,31,32,57].

DNS-over-HTTPS Analyzer (DoHlyzer) is an effective tool for extracting statistics-based features of DoH traffic [64]. The tool extracts features about DNS query and response statistics, including the number of DNS queries and responses, response time statistics, and query/response size statistics, as well as encryption-related metadata such as TLS handshake parameters and certificates. These features can be used to detect anomalies in DNS-over-HTTPS traffic through AI-based algorithms. Alzighaibi et al. [48] performed anomaly detection over encrypted traffic using features extracted using DoHlyzer.

- Log Information-Based Feature Extraction

Another feature extraction method frequently used in environments where useful information cannot be collected directly from packets due to encryption is to utilize logs generated by network equipment. Log data contain detailed information about system operations, events, errors, user actions, etc., and can be useful in cases where the actual information contained in the packet cannot be identified.

Zeek IDS [67,68] is an open source intrusion detection system and network analysis framework that analyzes network traffic in real time. This framework can define the capabilities that users want through scripted language, regardless of protocol, and generates detailed logs containing information about network activities, including statistics about connection information, DNS requests, HTTP transactions, and SSL certificates [5]. Zeek is a multipurpose tool that supports both signature-based detection methods and behavior-based anomaly detection methods to identify known threats and anomaly network behavior. Zeek IDS monitors the network and creates log files such as conn.log (connection information), x509.log (certificate information), and ssl.log (SSL/TLS information) for encrypted traffic. conn.log contains information such as protocol information, traffic volume statistics (total number of connections and number of bytes transferred), timestamp analysis (i.e., traffic patterns by hour, day), session duration, and port usage. x509.log contains information about encryption certificates, including certificate validity period, certificate authority (CA), and certificate usage. Finally, packet-level features such as packet size distribution and details about the SSL handshake can be obtained from ssl.log. Features collected or extracted from traffic encrypted by Zeek IDS are used in various anomaly detection research [5,11,19,22,32,43,54].

### 3.6. Feature Selections

The feature selection is an important process that identifies and selects the most relevant and informative features from a feature set. In a general machine learning context, if all collectible features are used to train a model, there is a possibility that the model will overfit to incorrect patterns inherent in the data. The feature selection improves the efficiency of machine learning and deep learning by adopting only some valuable features for model learning, reducing computational complexity and the risk of overfitting. Additionally, by improving the interpretability of the model, the reasons why specific features contribute to anomaly detection can be more easily explained. In other words, the feature selection improves the accuracy of the AI-based anomaly detection model by removing noise and duplication included in the dataset while allowing the model to focus on highly contributing information. We present a summary of frequently used feature selection methods to answer RQ3.

The Filtering method evaluates the relevance between features regardless of the type of machine learning algorithm or deep learning algorithm and selects useful features based on importance. Huo et al. [19] use the analysis of variance (ANOVA) method and mutual information (MI), which are representative filtering methods, to evaluate features and select features with high relevance for learning and anomaly detection. Li et al. [34] select features with high correlation through correlation analysis between features and use them for learning and anomaly detection.

Manual selection is a simple approach to selecting features based on expert intuition, domain knowledge, and requirements of a given problem. Many studies have removed features from encryption traffic that are not helpful for anomaly detection [39,42,48,50], at the authors’ judgment, and this feature selection method has also been employed in the process of standardizing data for use as an input for detection models or algorithms [12,39]. Wang et al. [43] experimented by dividing the features of one dataset into further optimized statistical (FOS) feature set, time-based feature set, tamper-resistant feature set, and side channel feature set based on the characteristics of the features.

Exhaustive Search is a method of systematically evaluating a subset of all possible features to identify the most appropriate feature combination for a specific machine learning task. The method starts by generating all possible subsets from the full set of features. This involves generating various combinations from a single feature to a subset containing all features. Wang et al. [43] extracted the top 10 ranked feature sets specialized for malware traffic detection and used them in experiments with the purpose of improving the performance of the anomaly detection model among the features of one dataset. The 10 features used in the experiment were extracted from the detection framework using the improved SFS algorithm [69].

### 3.7. Preprocessing

Data preprocessing refers to all processes that cleanse and transform raw data before starting data analysis or machine learning modeling. This process improves the quality of data and processes the data into a form suitable for analysis or modeling. Preprocessing typically includes missing value removal, scaling, outlier processing, and encoding. The raw network traffic contains many elements that are disadvantageous to model learning, such as noisy data and missing values. We present a summary of the characteristics and methods of frequently used preprocessing methods to answer RQ3.

Normalization aims to standardize the scale of values that features in a dataset can have. Normalization includes min-max normalization, which converts the range of different features in a dataset into a consistent range between 0 and 1 [6,70], and z-score, which converts the distribution of features into a standard normal distribution [5,55]. Normalization is necessary because many machine learning and deep learning algorithms are sensitive to the scale of input features. When features have different scales, features with larger ranges dominate the learning process, leading to biased models and potentially poor anomaly detection performance. By normalizing the features, each feature can contribute fairly to the anomaly detection task. This means that normalization can improve AI-based anomaly detection models’ stability, convergence, and accuracy, thereby improving their ability to detect anomalies.

Data cleaning is essential when the actual data have data quality problems such as missing values, outliers, and noisy observations. Data cleaning helps ensure the accuracy and reliability of the dataset, which is important for the effectiveness of anomaly detection models. Data cleaning not only improves the quality of datasets by processing missing values and outlier processing through incorrect data point removal techniques, correction techniques, and imputation techniques but also prevents these data with errors from distorting detection results, leading to false positives or negatives in the anomaly detection process. Ref. [42] removed unencrypted traffic from many flows, and ref. [43] removed network packets that were not relevant to the detection of encrypted malicious traffic, such as Address Resolution Protocol and Internet Control Message Protocol packets, as well as redundant, corrupt, unnecessary, or incompletely captured information. In [24,30,39], the authors excluded special information, such as SNI and some header information, which they believed interfered with the classification of normal and abnormal data.

Length unification aims to unify the length of variable-length data to the same length. To this end, techniques such as data padding or cutting are included. The padding adds a specific value (e.g., zero) to short data, and the cutting reduces the long sequence to match the desired length. In particular, network traffic is typical time series data, so preprocessing is essential. Length normalization is generally essential in machine learning and deep learning, where models require input of the same length in [12,21,24,30,54] the data length was normalized to 784 bytes for the purpose of transforming one-dimensional data into two-dimensional data, and [26,34,50] generated the same length of input data for network traffic data through normalization to a user-specified length and used it for anomaly detection experiments.

Data conversion encompasses the process of converting data from one format or representation to another representation suitable for analysis. By performing data conversion, it is converted into the input form required by the anomaly detection model. Since most machine learning and deep learning algorithms can only receive numeric data as an input, raw data need to be converted to numeric form. In addition, label encoding, and one-hot encoding methods are mainly used to convert categorical data into numerical values. Bakhshi et al. [6] used one-hot encoding to convert categorical data to numeric data and then performed anomaly detection. Additionally, a method of converting network packets into two-dimensional tensors is used for models that require two-dimensional data as input, such as a 2D convolutional neural network (CNN) [24,30,45,54]. In addition, a method of converting two-dimensional array data into images can be used to utilize AI models specialized for images for network traffic-based anomaly detection [24,26,30,36,39,54].

### 3.8. Detection Algorithm

There are various AI algorithms used for anomaly detection over encrypted traffic. We present a summary of the characteristics and capabilities of AI algorithms frequently employed in anomaly detection to answer RQ4. The frequency of each detection algorithms used to detect malicious and anomaly traffic derived by analyzing the literature is shown in Table 4.

Linear regression is the most basic machine learning model used to model the relationship between one or more independent variables (predictors) and a dependent variable (target) using linear equations. The linear regression is called simple regression analysis or multiple regression analysis, depending on the number of independent variables used to predict the dependent variable [71].

Logistic regression is mainly used to classify given data linearly [72]. The logistic regression, unlike the linear regression, was developed to deal with categorical data (i.e., the dependent variable is categorical).

Naïve Bayes (NB) is a probabilistic classification method based on Bayes’ theorem [73]. The biggest characteristic of naïve Bayes is that it assumes that the given features are independent of each other. This assumption has the effect of simplifying model implementation and reducing computational complexity. However, this model is not suitable for data with complex correlations, as features use a method of simplifying probability calculations under mutually independent assumptions. Additionally, depending on the distribution of the data assumed, there are various derivatives such as Gaussian NB or Bernoulli NB [74].

C4.5 is a model that develops the previous ID3 decision tree algorithm [75]. Notable improvements in C4.5 include handling continuous and discrete features, missing values, and pruning to avoid overfitting.

The Classification And Regression Tree (CART) is another variation of the decision tree algorithm and can be used for both classification and regression [76]. CART’s learning begins with feature selection and data segmentation. The most informative features are selected, and the data are divided into two subsets. This splitting operation is performed recursively and is repeated until no more information is obtained or the number of samples belonging to a node falls below a certain threshold. After tree growth is complete, overfitting of the model can be prevented through selective pruning.

The K-nearest neighbor (KNN) is a model that finds the k-nearest neighbors of a given data point and makes predictions based on their labels [77]. Therefore, KNN can classify without separate learning by simply measuring the distance between the training data and the newly given data at the time of prediction. At this time, various metrics such as Euclidean distance, Manhattan distance, or cosine similarity can be employed to measure the distance between data. There are also extended techniques, such as weighting important data in the training dataset.

Ensemble is a method of performing predictions on given data by simultaneously using several weak models rather than a specific algorithm or model and combining the results to improve the robustness of performance [78]. Generally, average ensembles that use the average of the prediction results of weak models are mainly used. Additionally, stacking is an ensemble learning technique that creates a meta-model by combining multiple classification or regression models [48].

Random forest is one of the ensemble learning methods and is constructed by combining several decision trees [79]. Random forest can also be used for classification, regression, and other machine learning tasks, and as an ensemble model, it is somewhat free from overfitting problems. Random forest is trained by learning each decision tree as a subset of the entire data. Additionally, because each decision tree is learned independently, parallel processing is possible.

The eXtreme gradient boosting (XGBoost) algorithm is a type of ensemble learning that combines multiple weak prediction models (usually decision trees) to form a strong prediction model [80]. Each tree is learned by correcting the errors of the previous tree. Similarly to gradient boosting, the model is updated at each step in a way that minimizes the gradient of the loss function. This model consumes significantly fewer resources than traditional predictive models by combining insights into cache access patterns, data compression, and sharding. In addition, XGBoost is an algorithm that can be learned in a distributed processing environment and takes CPU cache into account, so it can be learned at high speed even when the data size increases.

Support vector machine (SVM) is basically a classification algorithm that finds hyperplanes that separate data points. SVM is trained to maximize the margin between two classes. The k-SVM with the kernel trick maps data that cannot be linearly separated into a high dimension and then attempts linear separation in that space [81]. SVM shows very high classification accuracy for linearly separable data and can be extended to multi-class classification.

Light gradient boosting machine (LightGBM) is one of the gradient boosting frameworks and can be learned quickly even with large datasets and distributed computing environments [82]. LightGBM, similar to XGBoost, becomes a strong prediction model by sequentially learning multiple weak decision trees. However, unlike most tree-based algorithms that adopt a depth-wise growth approach, LightGBM adopts a leaf-wise approach, resulting in a more accurate prediction model. Additionally, because this model automatically processes missing values, separate preprocessing for missing values is not required.

Spatial–temporal graph (ST-graph) simultaneously models spatial location and temporal changes [46]. This graph expresses objects or locations as nodes and relationships between objects or locations as edges, and temporal information is given to each node and edge. ST-graph is used in various fields, such as traffic flow analysis, social network analysis, and environmental monitoring. These graphs enable the effective analysis of complex patterns and interactions across space and time. ST-graph can explore multiple features from a spatial and temporal perspective and integrate all available information for comprehensive malware traffic detection in cryptographic scenarios.

Deep forest (DF) is a model that learns complex patterns by stacking several layers of tree-based models. This algorithm uses the predictions of the tree model generated at each layer as input to the next layer, extracting progressively more complex features. DF can achieve high performance without the complexity of a neural network and is especially useful in problems where feature engineering is difficult or in small datasets. This algorithm maintains model interpretability along with high classification accuracy. DF-IDS is an intrusion detection algorithm created using DF [39].

Flow Sequence Network (FS-Net) is a recurrent neural network that models time series or sequential data [83]. This model learns complex patterns and interactions by considering the temporal flow of data. FS-Net generally uses multiple layers of recurrent networks and an attention mechanism to automatically extract temporal dependencies and important features. This structure is mainly used in various fields such as natural language processing, financial time series analysis, and sensor data analysis.

The adaptive boosting (AdaBoost) is a type of ensemble learning that learns a strong prediction model by combining several weak models [84]. This algorithm trains models sequentially and works by correcting the errors of the previous model at each step. AdaBoost assigns weights to each model, paying more attention to misclassified samples. This method can be applied not only to classification problems, but also to regression, and provides high accuracy and generalization ability.

Extremely randomized trees (Extra Trees) are ensemble learning methods that have a structure similar to random forest [85]. This algorithm is based on a decision tree but generates the tree more randomly to increase diversity. Extra Trees selects a random subset of features at each node but uses random partitioning instead of finding the optimal partition. This randomness improves the generalization performance of the model and prevents overfitting.

Multi-layer perceptron (MLP) is a method of sequentially attaching several layers of perceptrons [86]. Each layer acts like a node in the graph structure. In other words, when input comes in, calculation is performed and output is sent. KNN Graph-based MLP combines KNN and MLP to generate nodes of the attribute KNN Graph by using a MLP classifier [54]. The model can perform binary classification to determine whether an encrypted session is malicious.

Convolutional neural network (CNN) is a deep neural network model that can recognize and classify abstract features of images commonly used in computer vision [87]. CNN is mainly used in image classification, image processing, and object detection/segmentation and has recently shown excellent results in domains other than images, such as natural language processing and speech recognition. CNNs can be used in combination with other types of neural networks. For example, TCMal is a model that combines CNN and transformer [34]; ConvLaddernet is a combination of the ladder network and CNN [36].

Long short-term memory (LSTM) is an extension of the recurrent neural network (RNN) designed to avoid long-term dependency problems [37]. Unlike traditional RNNs, LSTMs can remember data for a long period of time. In a RNN architecture, the hidden layer (or cell) has a simple structure, while in LSTM, three gates (forget, input, and output gates) are introduced to better reflect the characteristics of time series data. Currently, LSTM has several variants: CBOW-LSTM, CBOW-BiLSTM, Skip-gram LSTM, and Skip-gram BiLSTM.

The gated recurrent unit (GRU) was designed to reduce the computational complexity of LSTM [88]. GRU uses update and reset gates that play similar roles instead of the three gates of LSTM. As a result, GRU can process time series data faster while maintaining similar performance to LSTM. GRU, like LSTM, has been derived in various ways. TLARNN is a model that combines bidirectional GRU and 1d-CNN and is used to extract spatial and temporal features of encrypted traffic packets [50].

K-means is one of the simplest clustering algorithms [89]. This algorithm uses distance (usually Euclidean distance) as a metric to classify a given dataset into k clusters.

A residual network (ResNet) is a type of neural network algorithm. General deep neural networks show a degradation problem in which prediction performance decreases as the number of layers increases. To overcome this problem, this model adds residual connections to transfer features from lower layers to higher layers and ensures that gradients are propagated efficiently in backpropagation. Yang et al. [45] used ResNet to detect anomalies with high accuracy from encrypted malicious traffic.

Efficientnet is a variation of CNN that optimizes both performance and efficiency [90]. This model adjusts the network depth, width, and input resolution by considering complexity and resource usage. Efficientnet uses a complex scaling method to achieve high performance with fewer parameters and computational amounts than other models. Due to these characteristics, Efficientnet shows excellent performance in various computing environments and is used for various machine learning tasks such as image classification, object detection, and object segmentation.

The stacked autoencoder (SAE) learns complex features by stacking multiple layers of autoencoders [26]. This model automatically extracts important characteristics of the data through the process of compressing and then restoring the input data. The autoencoder of each layer receives the output of the previous layer as input and gradually learns more complex features.

### 3.9. Performance Indicators

**Figure 3:**
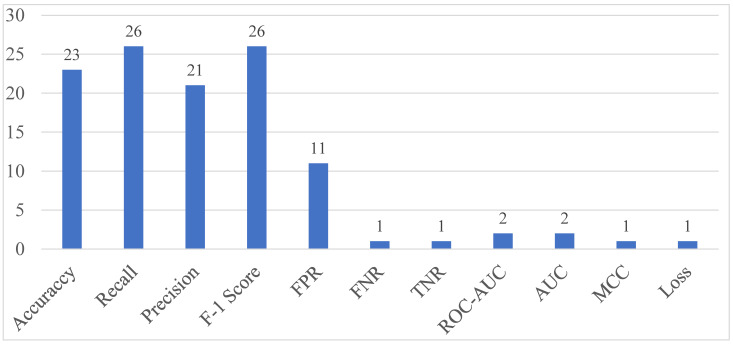
Performance indicators used in articles.

The performance indicators are quantitative indicators used to evaluate how well an anomaly detection model performs in various aspects. Figure 3 shows the frequency of performance indicators frequently employed in evaluating the performance of models de-rived by analyzing the literature. We describe the performance metrics used to answer RQ5 in the anomaly detection research on encrypted traffic and provide information on the frequency with which they were used.

Accuracy is an evaluation index that focuses on the overall prediction success of the detection system by calculating the ratio of correctly classified data to the total number of data. Accuracy was used as an evaluation index in 23 of the 32 articles analyzed. Accuracy can be used as an intuitive measure to evaluate system performance by providing performance evaluation results on how well normal and anomaly data are classified. However, accuracy is an evaluation index that focuses only on prediction success without considering the bias of data that occurs in the field. In other words, accuracy is not suitable for evaluating the performance of a detection model learned with a dataset containing less anomaly data than normal data.

$$Accuracy=\frac{TP+TN}{TP+FP+TN+FN}$$

Recall is an evaluation indicator that indicates the ratio of data that was correctly judged to be anomaly among data that was judged to be anomaly. Recall was used as an evaluation index in 26 of the 32 articles analyzed. Recall focuses on the system’s anomaly data detection function and enables the evaluation of the detection performance of anomaly data that are relatively sparse compared to normal data. In an environment where identification of anomaly data with very low observation frequency is important, recall is a very important evaluation index that helps the model respond sensitively and avoid potential risks.

$$Recall=\frac{TP}{TP+FN}$$

Precision is an evaluation index that indicates the ratio of actual anomaly data among data predicted as anomaly data. Precision is efficient in identifying false positives, which are cases where normal data are incorrectly classified as anomaly data. Precision was used as an evaluation index in 21 of the 32 articles analyzed.

$$Precision=\frac{TP}{TP+FP}$$

F1-score is an evaluation index that combines Precision and Recall to provide a balanced evaluation of the performance of the detection system and is suitable for evaluating the detection performance of the system by considering both false positives and false positives. F1-score is especially useful when evaluating models on imbalanced datasets where most of the data are normal. F1-score was used as an evaluation index in 26 of the 32 articles analyzed.

$$F1\;Score=\frac{2\times Precision\times Recall}{Precision+Recall}$$

The false positive rate (FPR) is an evaluation index that measures the ratio of normal data that are incorrectly classified as anomalies among all data points predicted as anomaly by an anomaly detection model. FPR is an evaluation index that evaluates the system’s ability to prevent false positives that occur when normal data are incorrectly classified as anomaly data. FPR was used as an evaluation index in 11 of the 32 articles analyzed.

$$FPR=\frac{FP}{FP+TN}$$

The true negative rate (TNR) is an evaluation indicator that indicates the rate of anomaly prediction of actual normal data and can be used when evaluating the performance of a model regarding false positive rates. TNR was used as an evaluation index in 1 of the 32 articles analyzed.

$$TNR=\frac{TN}{TN+FP}$$

The false negative rate (FNR) is an evaluation indicator that indicates the rate of prediction of actual anomaly data as normal and can be used when evaluating the performance of a model regarding the false negative rate. FNR was used as an evaluation index in 1 of the 32 articles analyzed.

$$FNR=\frac{FN}{TP+FN}$$

The receiver operating characteristic area under the curve (ROC-AUC), which is the area of the ROC curve according to the threshold, is an evaluation index that indicates the rate of change in the precision and FPR of the model. The value of ROC-AUC is expressed between 0 and 1, and the closer it is to 1, the better the model is at distinguishing between normal and anomaly data. ROC-AUC was used as an evaluation index in 2 of the 32 articles analyzed.

The Matthews Correlation Coefficient (MCC) is an evaluation index that evaluates the performance of a binary classification model by considering all confusion matrix values (TP, TN, FP, and FN). Because MCC considers the balance ratio of TP, TN, FP, and FN, it provides more information than F1-score and accuracy when evaluating binary classification problems. The higher the value, the better the model performance. MCC was used as an evaluation index in 1 of the 32 articles analyzed.

$$MCC=\frac{TP*TN-FP*FN}{\sqrt{\left(TP+FP)(TP+FN)(TN+FP)(TN+FN\right)}}$$

Loss generally refers to the error or loss that occurs in machine learning and deep learning model training. In general, mean squared error (MSE) and mean absolute error (MAE) are mainly used as loss functions for regression problems, and loss functions such as cross entropy are used for classification models. The lower the loss value, the more accurately the model predicts. Loss was used as an evaluation index in 1 of the 32 articles analyzed.

## 4. Discussion

In order to conduct a systematic literature review, this article set up questions about datasets, feature extraction methods, data processing technologies, AI algorithms for anomaly detection, and performance evaluation indicators to evaluate them in Section 2.1. This chapter provides answers to this.

### 4.1. RQ1: What Datasets Are Mainly Used to Measure the Performance of Anomaly Detection Models over Encrypted Traffic, and What Encryption Method Was Mainly Used to Encrypt the Data Used in the Experiment?

To answer RQ1, in Section 3.4, we listed datasets frequently used in AI-based anomaly detection research targeting encrypted traffic and summarized the characteristics of each dataset. The literature analysis shows that most datasets do not consist only of encrypted traffic but are a mixture of different types of packets and traffic, including unencrypted traffic. For this reason, many research studies extract traffic encrypted with a specific protocol from a dataset and use the extracted traffic dataset as the original dataset to train an anomaly detection model. Additionally, there have been cases where encrypted traffic extracted from multiple datasets was combined to ensure diversity in cyber-attacks and adjust the ratio of normal/anomaly data. Most research used encrypted traffic generated by TLS and SSH.

However, most of these encrypted traffic datasets were created based on data collected in general IT environments, and most AI-based anomaly detection research studies targeting encrypted traffic were conducted on general IT environments due to limitations of the datasets. Recently, the frequency of cyber-attacks against special environments such as industrial control systems (ICS) and defense weapon systems is increasing, and the types of attacks are also becoming more diverse. For this reason, ICS and defense weapon systems are also using encrypted communications, but cyber-attacks still occur. In order to detect such cyber-attacks, AI-based anomaly detection research targeting ICS and weapon systems must be conducted. However, there are difficulties in conducting AI-based anomaly detection research due to the absence of datasets created and collected in the ICS and defense weapons system environment. To solve this problem, it is essential to create a dataset for the ICS environment and weapon system, and research should be conducted to create a dataset for the data generated in this special environment.

### 4.2. RQ2: How Can the Features Needed for AI Model Learning and Anomaly Detection over Encrypted Traffic Be Extracted?

To answer RQ2, we summarize frequently used feature extraction methods and their characteristics in AI-based anomaly detection research over encrypted traffic in Section 3.5. The point of encryption is to protect data from unauthorized access. This means that the data within the encrypted packets are intentionally randomized, making analysis difficult. For this reason, the method of utilizing important information as a function by applying DPI to encrypted traffic cannot be applied. As an alternative, anomaly detection can be proposed by decrypting encrypted traffic, but it is virtually impossible to decrypt encrypted data without a decryption key. Additionally, this method is not recommended for detecting anomalies where real-time is important because there is a risk of exposing important information during the decryption process and a risk of performance degradation such as network traffic bottlenecks. For this reason, most research extracted the characteristics of encrypted traffic through statistical processing for identifiable data in encrypted traffic, and features such as source/destination port, duration, packet time change, and average packet time were often used. In addition, a method of using key information values of log files generated from network equipment that monitors traffic as features was adopted, and features such as average duration of connection, ratio of SSL connections, and number of inbound packets were mainly used. It is judged that the effectiveness of the study using the anomaly detection method using this feature extraction method has been verified as the high-performance results were derived. For this reason, it is expected that the method of extracting features from encrypted traffic for AI-based anomaly detection will continue to use packet metadata and statistical characteristics.

### 4.3. RQ3: What Data Preprocessing Technology Is Used to Detect Malicious Activity Using Encrypted Traffic?

To answer RQ3, we presented the feature selection and preprocessing techniques used in our study of AI-based anomaly detection targeting encrypted traffic in Section 3.6 and Section 3.7, respectively. As a result of the literature analysis, feature selection methods such as filtering method, manual selection, and exhaustive search were mainly used in this research field. Additionally, normalization, data cleaning, length unification, and data transformation have been widely used as preprocessing techniques. In most research, the length unification method was applied to inject data of consistent size for the purpose of improving the performance of anomaly detection models. In addition, dimensional transformation and image scaling were used to satisfy the input requirements of algorithms that require input of two-dimensional data and image data, such as 2D-CNN. This data processing method is also frequently used in research on anomaly detection over plaintext traffic. The difference in data processing methods between the AI-based anomaly detection method for encrypted traffic and the AI-based anomaly detection method over plaintext traffic that is currently being researched is not big.

In addition, it was confirmed that most studies do not consider real-time processing methods for data processing and focus on offline or batch processing. This may not be practical in fields where a lot of traffic is generated, and data are required to be processed in real time. Real-time processing of data is a very important function when implementing an AI-based anomaly detection system and applying it to the field. For this reason, research should be done on real-time processing of data.

### 4.4. RQ4: What Is the AI Algorithm Used to Detect Anomalies in Encrypted Traffic?

To answer RQ4, Section 3.8 lists the detection algorithms used in AI-based anomaly detection research on encrypted traffic and summarizes the characteristics of each AI algorithm. As a result of the literature analysis, a variety of detection algorithms are used, including traditional machine learning algorithms and deep learning-based algorithms. Table 4 shows that for machine learning-based anomaly detection algorithms, random forest is traditionally the most popular machine learning algorithm, followed by XGBoost, decision trees, naive Bayes, and ensembles. As deep learning-based anomaly detection algorithms, CNN algorithms such as 1-D CNN and 2-D CNN are most frequently used, followed by autoencoder, LSTM, FS-Net, and GRU. AI-based anomaly detection research over encrypted traffic use both supervised and unsupervised learning algorithms. However, it was confirmed that there are more research using super-vised learning-based algorithms for detailed classification of cyber-attacks. Supervised learning-based algorithms specialize in classifying labeled attacks, making it difficult to detect anomalies for new types of cyber-attacks, such as zero-day attacks. Because most datasets included labeled normal traffic and abnormal traffic, it was possible to apply the supervised learning algorithm. However, in a real environment, not only are there difficulties in collecting attack traffic, but also anomaly detection performance may be affected due to differences in labeling methods for data. For this reason, it is expected that more research on unsupervised learning will be presented. In general, algorithms in unsupervised learning attempt to find relationships between features without labels, which is a suitable feature for finding unknown attacks. However, in a network environment where assets are rapidly changing and added, if rapid model updates are not made, unsupervised learning-based algorithms may encounter problems such as detecting normal traffic as anomaly traffic. Therefore, an anomaly detection algorithm must be selected considering the environmental factors of the infrastructure to which the AI-based anomaly detection model will be applied. Future research should not only consider anomaly detection performance, but also proceed in the direction of applying an appropriate anomaly detection algorithm by considering environmental factors of the infrastructure to which the anomaly detection model will be applied.

### 4.5. RQ5: What Are the Performance Indicators Used to Evaluate the Performance of an Anomaly Detection Model over Encrypted Traffic?

To answer RQ5, we summarize in Section 3.9 the performance metrics used to evaluate the performance of AI-based anomaly detection models over encrypted traffic in the analyzed literature. The literature analysis showed that recall and F-1 score metrics were most frequently used, followed by accuracy, precision, and FPR. Detection performance metrics such as MCC, loss, FNR, ROC-AUC, AUC, and TNR were also used to evaluate the anomaly detection performance. Figure 3 shows the frequency of performance metrics used in the studies. Table 2 also specifies the performance indicators used in each analyzed research. Each research used various performance evaluation indicators to analyze the anomaly detection performance of the anomaly detection model from various perspectives. In most research, the performance of anomaly detection models was evaluated using only the anomaly detection performance evaluation indices of the anomaly detection model. However, when an anomaly detection model is applied to the field and anomaly detection is performed on encrypted traffic generated in real time, various considerations are needed not only in terms of detection performance but also in the operational aspect of the anomaly detection model. These considerations include computing efficiency and anomaly detection time.

Computing efficiency is an evaluation indicator of how little an anomaly detection model uses resources such as CPU, memory, and GPU during the period of anomaly detection for encrypted traffic. When an anomaly detection model is applied to the field and anomaly detection is performed, anomaly detection must be performed in real time on the encrypted traffic that occurs in countless quantities. For this reason, the anomaly detection model must continuously perform many calculations. If the computing efficiency of the anomaly detection model is low, it may overload computing resources. Overload of computing resources can adversely affect detection performance and anomaly detection time, so it is a factor that must be considered when developing an anomaly detection model over encrypted traffic.

Anomaly detection time refers to the time it takes to analyze encrypted traffic and classify whether the traffic is abnormal or not. If the anomaly detection time is high, it means that the algorithm complexity of the anomaly detection model is high. When an anomaly detection model is introduced into the actual field, high algorithmic complexity not only burdens computing resources but also has the negative impact of delaying anomaly detection time. Anomaly detection time can be reduced by selecting an appropriate anomaly detection algorithm and selecting an appropriate dataset preprocessing method and is a factor that must be considered when developing an anomaly detection model for encrypted traffic.

For this reason, research should be conducted that considers not only the anomaly detection performance of the anomaly detection model, but also the performance of the anomaly detection model in terms of operation, such as computing efficiency and anomaly detection time.

## 5. Conclusions

In modern society, encrypted communication methods must be applied to protect information and systems. However, as the types of hidden cyber-attacks exploiting encrypted communications are becoming more diverse and the frequency of cyber-attacks is increasing, AI-based anomaly detection research over encrypted traffic has become a research field that must be investigated.

In this review, we conducted a systematic review of the related literature to provide a discussion of trends and future research in AI-based anomaly detection technology over encrypted traffic that is currently being researched. This review was conducted on 32 articles on AI-based anomaly detection research over encrypted traffic. In order to review research on AI-based anomaly detection technology for encrypted traffic from various perspectives such as dataset, feature extraction, feature selection, preprocessing, anomaly detection algorithm, and performance indicators, related research questions are set, and a discussion is provided.

As a result of the literature analysis, it was confirmed that AI-based anomaly detection research on encrypted traffic occurring in various environments is not being conducted smoothly. This is believed to be due to the absence of encrypted traffic datasets collected in various environments. Most research studies have confirmed that a high performance of anomaly detection models over encrypted traffic has been achieved. However, the data processing methods and anomaly detection model performance evaluation methods used in most research have not fully included operational and environmental considerations for the environment in which AI-based anomaly detection models over encrypted traffic will be applied.

In future studies, AI-based anomaly detection research over encrypted traffic, operational and environmental considerations for the environment in which the anomaly detection model will be applied should be identified, and research taking these into account should be conducted.

## Figures and Tables

**Table 1:** Search query for bibliographic databases.

Bibliographic Database	Search Queries
Web of Science (# of results: 56)	(ALL = (encrypted) AND ALL = (network) AND ALL = (traffic) AND ALL = (anomaly or anomalies) AND ALL = (detection or detecting)) AND (PY == (“2023” OR “2022” OR “2021” OR “2020” OR “2019”))
Scopus (# of results: 89)	(TITLE-ABS-KEY (encrypted) AND TITLE-ABS-KEY (network) AND TITLE-ABS-KEY (traffic) AND TITLE-ABS-KEY (detection OR detecting) AND TITLE-ABS-KEY (anomaly OR anomalies)) AND PUBYEAR > 2018 AND PUBYEAR < 2024
ACM (# of results: 121)	AllField:(encrypted) AND AllField:(network) AND AllField:(traffic) AND Title:(anomal*) AND AllField:(detect*) “filter”: {E-Publication Date: (1 January 2019 TO 31 August 2023)}
IEEE Xplore (# of results: 60)	(“All Metadata”:encrypted) AND (“All Metadata”:network) AND (“All Metadata”:traffic) AND (“All Metadata”:anomal*) AND (“All Metadata”:detect*) (Publication Year: 2019–2023)

**Table 2:** Summary of the identified literature.

Article	Dataset	Encryption Protocol	Feature Extraction	Feature Selection	Preprocessing	Classification Algorithm	Performance Metrics
Chen et al. [5]	Mixed (CTU-Malware-Captures [9], MTA dataset [10])	SSL/TLS	Log Information-Based Feature Extraction	-	Normalization (−1,1)	XGBoost, SVM, Random Forest	Accuracy, F1-score
Chao et al. [11]	CTU-Malware-Captures [9]	SSL/TLS	Log Information-Based Feature Extraction	-	-	LightGBM	Accuracy, Precision, Recall, F1-score, FPR, FNR, TNR,
Chen et al. [12]	ISCX VPN-NONVPN [13], USTC_2016 [14], Self-collected data in the power system environment	SSL, SFTP, FTPS	-	Select only the first 784 bytes of the session to use as a feature	Length unification, Convert to two-dimensional data, Converted to 2D grayscale images	1D-CNN, 2D-CNN	Precision, Recall
Bakhshi et al. [6]	NSL-KDD [15]	SSH	Statistics-based feature extraction	-	Normalization (0,1), One-Hot Encoding	CNN, LSTM, GRU, CNN+GRU	Accuracy, Precision, Recall, FPR, F1-Score
	UNSW-NB15 [16]	TLS, SSH					
	CIC-IDS-2017 [17]	TLS, SSH,					
Garcia et al. [18]	Self-collection Slow DoS dataset	TLS	Directly implemented Conversation Processor	-	Normalization	Autoencoder	Accuracy, Precision, Recall, FPR, F1-Score
Huo et al. [19]	CTU-13 [20]	TLS	Log Information-Based Feature Extraction	Analysis of variance (ANOVA) method and mutual information (MI)	-	Random Forest, XGBoost, GNB	Accuracy, Precision, Recall, F1-score, FPR
Yang et al. [21]	CTU-Malware-Captures [9]	TLS	-	Select only the first 784 bytes of the session to use as a feature	Length unification, data cleaning	ResNet	Accuracy, Precision, Recall, F1-score, MCC
Zhoa et al. [22]	Datacon 2020 Dataset [23]	TLS	Log Information-Based Feature Extraction	-	-	Ensemble (RF, NB, TEXTCNN)	Recall, FPR
Zhang et al. [24]	Mixed (ISCX VPN-nonVPN [13], CTU-13 [20])	SSL/TLS	Statistics-based feature extraction	-	Data cleaning, Length unification, Converted to 2D grayscale images	Efficientnet	Accuracy, Precision, Recall, F1-score
Lucia et al. [25]	CTU-13 [20]	TLS	Statistics-based feature extraction	-	-	SVM, 1D-CNN	Accuracy, Precision, Recall, F1-score, FPR
Zeng et al. [26]	Mixed ISCX VPN-nonVPN [13], ISCX 2012 IDS [27])	SSL, HTTPS	-	-	Package Generation, Traffic Purification, Traffic Refiner, Length unification	1D-CNN, LSTM, SAE	Precision, Recall, F1-score
Han et al. [28]	Datacon2020 Dataset [23]	TLS	Statistics-based feature extraction	-	-	Autoencoder	Accuracy, Precision, Recall, F1-score
Zhao et al. [29]	USTC-TFC2016 [14]	TLS	Statistics-based feature extraction	-	-	ERNN	Accuracy, F1-score
Wang et al. [30]	ISCX VPN-nonVPN [13], CICAndMal2017 [31]	VPN, TLS	Statistics-based feature extraction	-	Length unification, Convert to two-dimensional data, Converted to 2D grayscale images	2D-CNN	Accuracy, Precision, F1-score
Niu et al. [32]	Mixed (MTA dataset [10], MCFP dataset [33], CTU-13 [20])	TLS	Log Information-Based Feature Extraction	-	-	Improved Adaptive Random Forests	Precision, Recall, F1-score
Li et al. [34]	MTA dataset [10], STRA dataset [35], USTC-TFC2016 [14]	-	Statistics-based feature extraction	Correlation analysis	Length unification	TCMal (Transformer Encoder, CNN)	Accuracy, Precision, Recall, F1-score
Liu et al. [36]	ISCX VPN-nonVPN [13], USTC-TFC2016 [14]	SSL/TLS	Statistics-based feature extraction	-	Convert to two-dimensional data, Converted to 2D grayscale images	ConvLaddernet (CNN, Ladder network)	Accuracy, Precision, Recall, F1-score
Andrey et al. [37]	Mixed (CTU-Malware-Captures [9], Jason Stroschein’s public GitHub malware dataset [38])	TLS	Extract TLS session capability from raw Pcap file	-	Convert TLS session extraction words to 300-dimensional vectors	CBOW-LSTM, CBOW-BiLSTM, Skip-gram LSTM, Skip-gram BiLSTM	F1-score
Zhang et al. [39]	Mixed (MCFP dataset [33], ISCX VPN-nonVPN [13]), MTA dataset [10]	SSL/TLS	Statistics-based feature extraction	Traffic processing: removes special information that prevents classification (SNI, packet header) and extracts only the first N bytes of the session	Convert to two-dimensional data, Converted to 2D grayscale images	DF-IDS (XGBoost, Random Forest, Extra Trees)	Recall, FPR
Zheng et al. [40]	Datacon 2020 Dataset [23]	SSL/TLS	Statistics-based feature extraction	-	-	Linear Regression, BernoulliNB, Decision Trees, XGBoost, GCN-TC, GCN + XGBoost, GCN + Random Forest, GCN + KNN, GCN + DT, GCN-ETA	Accuracy, F1-score, AUC
Zhang et al. [41]	CTU-Malware-Captures [9]	TLS	MEMTD translates raw traffic into TLS, HPB, PLS, PAIS, extracting that information into features	-	-	Contextual LSTM, FS-Net, FusionNet	F1-score
Li al. [42]	CTU-Malware-Captures [9]	SSL/TLS	Statistics-based feature extraction	Delete features unrelated to classification, such as Ip and Port	-	1D-CNN, 2D-CNN	Accuracy, Precision, Recall, F1-score
Wang et al. [43]	Mixed (UNSW NS 2019 [44], CICIDS-2017 [17], CIC-AndMal 2017 [31], MCFP dataset [33], CICIDS-2012 [27])	SSL/TLS	Session-based feature extraction, Log Information-Based Feature Extraction	Select the features that fit the purpose of the five feature sets	-	Random Forest, KNN, CART, C4.5, MLP, NB, XGBoost, AdaBoost, Linear Regression, Logistic Regression,	Accuracy, Roc-AUC, Recall, FPR
Bader et al. [45]	Mixed (STRA dataset [35], ISCX VPN-non-VPN [13], MTA dataset [10])	TLS	Generating session data for 32 TLS packets and then generating feature information	Select features and statistics in a TLS session	Generate a matrix (5 × 4) for 14 features of 5 TLS packets	1D CNN, 2D CNN, Random Forest, SVM, KNN	Accuracy, Precision, Recall, F1-score
Fu et al. [46]	CICInvesAndMal 2019 [47], EncMal2021(Self-collection)	SSL/TLS	Statistics-based feature extraction, Extract TLS information and DGA-related features	-	-	Random Forest, FS-Net, ST-Graph	Precision, Recall, FPR
Ahmad et al. [48]	CIRA-CIC-DoHBrw-2020 [49]	Https	Statistics-based feature extraction	-	Chi-square filtering (features with similarly non-numeric values are replaced with numeric values using the same chi-square filtering algorithm), Replace missing values (determination of valid values)	Stacking (Random Forest and Decision Tree)	Accuracy, Precision, Recall, F1-score
Liu et al. [50]	CICAndMal2017 [31]	TLS	Statistics-based feature extraction	Ethernet head removal, IP address masking	Length unification	TLARNN (1D-CNN, biGRU)	Accuracy, Precision, Recall, F1-score
Wang et al. [51]	Mixed (CTU-Malware-Captures [9], CTU-Normal-Captures [52], CTU-Mixed-Captures [53], CICIDS-2017 [17], CICIDS-2012 [27], CIRA-CIC-DoHBRW-2020 [49])	SSL/TLS	Statistics-based feature extraction	-	-	Random Forest, Average Ensemble	Accuracy, Precision, Recall, F1-score ROC-AUC, FPR
Hong et al. [54]	Mixed (MCFP dataset [33], CTU-13 dataset [20])	TLS	Log Information-Based Feature Extraction	-	Length unification, Convert to two-dimensional data Converted to 2D grayscale images	KNN Graph-based MLP	Accuracy, Precision, Recall, F1-score
Abhay et al. [55]	MCFP dataset [33]	HTTPS	Statistics-based feature extraction	-	Numericalization, Data Cleaning, Data Normalization	Random Forest, Decision Tree, Extra trees, AdaBoost	Accuracy, Precision, Recall, F1-score, Model building time, Detection time
Xing et al. [56]	Mixed (CTU-13 [20], STRA dataset [35])	SSL/TLS	Statistics-based feature extraction, Sequential Features Extracting	-	-	LSTM-based Autoencoder, Deep dictionary learning	Precision, Recall F1-score
Bahlali et al. [57]	UNSW-NB15 [16], CSE-CIC-IDS2018 [58]	HTTPS, SSH, TLS	Statistics-based feature extraction	-	-	Autoencoder	Accuracy, Precision, Recall, FAR, F1-score

**Table 3:** The number of identified research, by year of publication and reference types.

Year of Research Publication	The Number of Selected Research
2019	2
2020	4
2021	5
2022	12
2023	9
**Reference type**	
Journal	18
Conference proceedings	14

**Table 4:** Detection algorithm used in articles.

Type	Detection Algorithm	Count
Machine Learning	Random Forest	7
	XGBoost	4
	Decision Trees	3
	Naïve Bayes (NB)	3
	Ensemble	3
	SVM	2
	Extra Trees	2
	Linear Regression (LR)	2
	KNN	2
	AdaBoost	2
	LightGBM	1
	K-means	1
	Improved Adaptive Random Forest	1
	Logistic Regression	1
	CART	1
Deep Learning	CNN	10
	Autoencoder	5
	LSTM	4
	FS-Net	2
	GRU	2
	ResNet	1
	Efficientnet	1
	Transformer Encoder	1
	Error-Resilient RNN(ERNN)	1
	Ladder network	1
	FusionNet	1
	ST-Graph	1
	Deep dictionary learning	1
	Multi-Layer Perceptron (MLP)	1

## Data Availability

Publicly available data and the corresponding URLs are provided in the text.
